# Mental Skills Training: An Often-Overlooked Aspect of Preparation for Future High-Performing Athletes in Sports Schools

**DOI:** 10.3390/bs16071109

**Published:** 2026-07-03

**Authors:** Sebastian Schröder, Christine Stucke, Tabea Linkohr, Melanie Schulz

**Affiliations:** 1 Department III Philology, Philosophy, Sports, Otto-von-Guericke-Universität, Magdeburg 1, 39104 Magdeburg, Germany; 2 Faculty of Culture, Media, Psychology, Macromedia University of Applied Science, 04105 Leipzig, Germany; 3 Leichtathletik-Verband Sachsen-Anhalt e. V., 06120 Halle, Germany

**Keywords:** self-efficacy beliefs, elite sports schools, achievement motivation, achievement motives

## Abstract

The present study aims to analyze the development of achievement motivation and self-efficacy belief in the context of elite sports schools. A total of 658 athletes (349 female, 309 male) from Year 5 onwards participated in the central trials and performance assessments in track and field for elite sports schools between 2016 and 2025. In addition to the analysis of physical and athletic performance, the following aspects were also documented: achievement motivation, need for achievement motives and general self-efficacy beliefs. Firstly, differences between the genders were measured in terms of fear of failure and confidence, exhibiting a small effect size ranging from 0.175 to 0.25 and a significance of 0.001 and 0.026. A subsequent analysis of the Kruskal–Wallis test, pertaining to the various groups with differing performance levels, revealed significant disparities in self-discipline (*p* = 0.010), goal setting (*p* = 0.013) and confidence (*p* = 0.029). The effect sizes for these differences ranged from 0.08 to 0.14, indicating a modest magnitude of impact. The psychological profile of the top athletes, which is based on the psychological determinants of the study, differs significantly from that of the other groups of athletes at time t_1_ (*p* = 0.001). It is recommended that appropriate training and guidance from coaches and sports psychologists be provided, given that confidence and self-efficacy expectations are key predictors of physical and athletic performance.

## 1. Introduction

Successfully supporting talented young athletes primarily involves talent scouting, talent retention and optimal talent development. Optimal support during school years is therefore a significant factor in nurturing young sporting talent. Elite sports schools (ESS) aim to create optimized conditions and in so doing have a positive influence on career development.

Attending an elite sports school brings significant changes to athletes’ everyday lives, making self-competence a key factor in successfully adapting to this new environment. The development of new strategies for stress management could be a key factor in achieving success.

As Wolfarth (2026) has stated, there has been a notable increase in mental health issues in recent years. The support team of the German Olympic Team now includes Welfare Officers. These psychologists have undergone specialist training and are the designated liaison officers for athletes and support staff alike.

The IOC is committed to ensuring the well-being of athletes beyond the competition period. In 2023, the IOC launched its Mental Health Action Plan, a comprehensive roadmap designed to promote mental well-being in sport.

How can we support mental skills in early age? In order to demonstrate long-term success, it is essential to measure it longitudinally. This illustrates the considerable impact of a robust school structure, which is fundamental to sporting success, particularly within the framework of elite schools of sport (ESS).

### 1.1. Dual Career at the Elite Schools of Sport (ESS)

Combining school attendance and an elite sports career inevitably leads to major challenges in terms of time management, stress and overcoming system-specific peculiarities (Stambulova & Wylleman, 2019; Henriksen et al., 2020; Kiens & Larsen, 2021, Stambulova & Harwood, 2022). Sports psychology support programs can help students to develop strategies for overcoming these challenges, thus positively influencing their careers (Pummell & Lavallee, 2019; Schröder, 2026). In their 2012 study, Borggrefe and Cachay (2012) examined the various stress factors experienced by students at an elite sports school, addressing these factors across multiple dimensions. The authors discuss social dimensions that take human resources into account, providing support in areas such as organization, learning and psychological issues. The temporal dimension is intended to allow for increased flexibility, particularly in the organization of school and lessons.

The dual mission of school sports: Education for and through sport also applies to elite sports schools in the German education system. The protection of children’s rights and welfare is the responsibility of every individual and institution that comes into contact with children and young people, especially those responsible for their care and supervision in kindergartens, after-school care centers, schools, leisure facilities, and health and social services (Gharwal, 2023). These moral principles must be reconciled with the demands of competitive sport, which place great physical and mental strain on young athletes. The inclusion of mental skills in the selection process and their early promotion is therefore a basic prerequisite for selecting and promoting young talents to best effect. The early promotion of mental skills is currently underrepresented, whereas the inclusion of physiological parameters has been studied extensively. Mental exhaustion could also play a role, especially in the context of a varied training program.

Güllich (2012) conducted a study to investigate the effectiveness of support systems, comparing two groups of athletes, one very successful and one less successful. The present study examined the characteristics that distinguish the two groups, and the correlations between early sporting success and subsequent career development. The results were collected in a cross-sectional study of 1558 athletes (57% male, 43% female) and examined in a second longitudinal study (*N* = 119) over three years. The results confirm the hypotheses formulated above that a varied training program for athletes has a positive effect on performance development in elite sport. Early specialization counteracts positive athletic development. A varied athletic training program is not at odds with the extensive training required by professional associations in their framework training plans. Rather, versatile basic training leads to an increase in the scope of training and thus to an enrichment of sporting experience, which on the one hand broadens the spectrum of coordinative abilities and on the other hand offers injury prevention, as a variety of movement programs are learned. It is worth observing that no mention is made of the connection between volume-oriented sports and the demand for versatile training. According to international standards, young swimmers are required to train for approximately 6 h per day. Considering the demands of school, the demand for more extensive sports training seems utopian. The German Swimming Association, for example, requires athletes in the second stage of training, i.e., schoolchildren aged 11 to 14, to swim approximately 60 km per week (Rudolf, 2015). If we add in versatility-oriented content, pre-pubescent swimmers end up training for 7 h or more per day.

As mentioned above, physical development characteristics are often ascribed greater importance than psychological parameters when it comes to talent scouting. MacNamara et al. (2010) highlighted the intricacies of the talent development process, emphasizing the potential influence of psychological factors on an athlete’s ability to convert potential into elite performance. It is vital to identify the motivational prerequisites in order to ensure the success of the project. Hoffmann (2005, p. 48) outlines the following motivational parameters: enjoyment of exercise, improvement of one’s own performance, togetherness, group dynamics (shared interests with other athletes, friendships), success, personal bests, aesthetics (body awareness) and athleticism (strength, flexibility, fitness, coordination).

The issue is to determine how the factors of joyful participation in sport described by Hoffmann (2005) can be integrated into everyday school sports while ensuring sufficient time is allocated to the respective sport.

### 1.2. Dropout

Determining precise figures on the rate of children and young people dropping out of competitive sports is challenging. This is due to the fact that these rates are dependent on various factors, including the specific sports, the financial and material support available, the infrastructure in place, and the education system of the respective country. However, a high rate of attrition among adolescents has been observed in many studies. For instance, 85% of young Slovenian athletes abandon athletics as they reach the senior age group, irrespective of their chosen discipline. Møllerløkken et al. (2015) described that approximately 25% of adolescents aged between 10 and 18 years drop out of football on an annual basis. A higher rate of player attrition was also observed among young Turkish soccer players, with 50.6% of players dropping out in the 2016–2017 season (Dut et al., 2019). In a longitudinal study of German swimmers, Staub et al. (2020) found that only 33% of those ranked among the top 100 at the age of 11 were also ranked in the top 100 at the age of 18.

In light of the potential adverse consequences of dropping out of elite sports during childhood and adolescence, there is an ongoing need to acquire more knowledge about the factors influencing such decisions. This knowledge can then be used to develop preventive strategies and to create environments that support young people’s adherence to elite sports (Back et al., 2022). A number of systematic reviews have been published which synthesize the research on factors influencing dropout from elite sport in young people across different sports.

In a review of nine studies, Múrcia Corrales and Olaya-Cuartero (2022) concluded that a close environment and a good relationship with the trainer are key factors in avoiding dropout in endurance sports. Another systematic review by Monteiro et al. (2017) highlighted that the presence of parents or coaches was identified as a factor that may contribute to high dropout rates in swimming. A lack of enjoyment and discouragement were also cited as key determinants of dropout rates in this sport (Blecharz et al., 2015). Moulds et al. (2024) reported in their systematic review that relative age, sex, competence perception, parental socio-demographics, and conflicts with other activities are factors frequently identified as influential to dropout.

A study by Orosz and Mezo (2015) showed differences between the most talented football players and three other groups in terms of psychological variables, with the most talented football players performing significantly better in pressure situations (*p* = 0.009) and freedom from worry (*p* = 0.001). Self-efficacy and self-concept showed differences between the groups, with the more talented group showing better results. Another piece of research has indicated that high-performing athletes tend to view elevated levels of stress as a beneficial challenge, while other athletes of similar age and skill level often perceive the same conditions as stressful (Schröder, 2026).

Creating detailed profiles of successful Olympic squads should provide information on the extent to which these correspond to talents at the time of selection and what measures may need to be taken to promote the best possible profiles. Therefore, this paper creates profiles of top athletes that may serve as a template for future talents, emphasizing that psychological factors are just as crucial as physiological indicators for a successful career (Harwood et al., 2004, McNeill & Wang, 2005, Schröder, 2025b). In particular, achievement motivation, need for achievement motives and self-efficacy beliefs are crucial in this context for ensuring a stable career.

## 2. Methodology

A total of 658 athletes (349 female, 309 male, mean age_(t1)_ = 11.42, SD = 1.32) from Year 5 onwards participated in the central trials and performance assessments in track and field for the ESS (Halle/Magdeburg) between 2016 and 2025. In addition to analyzing physical and athletic performance, aspects of achievement motivation (SMT, Olofsson et al., 2008), need for achievement motives (AMS Sport, Elbe et al., 2005) and general self-efficacy beliefs (Jerusalem & Schwarzer, 1999) were also recorded. For this reason, data measurement was an integral part of the annual October talent recruitment process. The number of athletes in each testing group is subject to fluctuation due to the number of individuals who did not complete the questionnaires in full.

**Figure 1:**
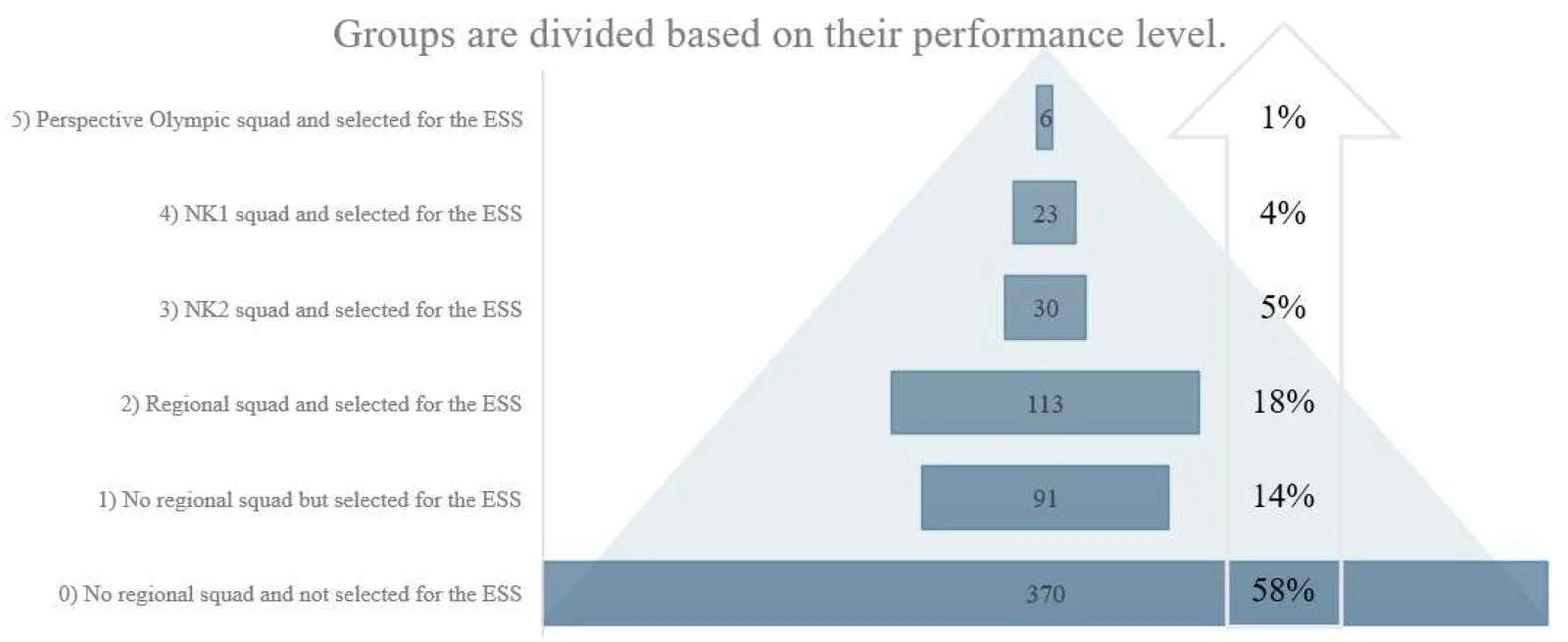
Categorization of individuals based on performance levels.

The athletes’ success levels were classified based on their respective squad status (*N* = 633). This classification enabled the formation of groups that could be divided into athletes of the following categories, although the data for 25 athletes were inconsistent (see Figure 1). The regional squad status is for high-performing athletes compared on a regional basis, the NK2 squad classifies the best-performing athletes at regional level who will be prepared for the national levels, the NK1 squad classifies athletes of higher national standards and the prospective Olympic status contains the best athletes nationally.

The groups were coded from 0 to 5 and possible group differences were then calculated using the Kruskal–Wallis test and SPSS 21. This is therefore a cross-sectional study that compares the different groups (0–5) in terms of their results at t_1_. Despite the fact that there are six groups in total, groups 5 and 6 were combined to form a larger group sample, thus avoiding any potential statistical inaccuracies resulting from the inclusion of the PK athletes within a group of six athletes (see Table 4).

Finally, profiles of the respective groups were created in order to be able to use a possible success profile of future top athletes as a guide for talent identification at t_1_. The Chi-squared test (*χ*^2^ test) was used to analyze group profiles (i.e., the distribution patterns of categorical data) for significant differences. This approach can be employed in aptitude testing, for example, to identify the best possible matches with the requirement profile of a given sport. In the event that the test scales within a profile are standardized (e.g., standard scores) and the reliabilities are known, changes in or comparisons of profiles can be examined using the Chi-squared distribution of profile scores (Pospeschill, 2025). Amelang and Zielinski (2004), for instance, used this method in the context of vocational aptitude testing. The key variables observed in the study are outlined in the following sub-categories: SMT endurance, flexibility, self-discipline, goal setting and confidence. For the achievement motive scale, the hope for success (HfS) and the fear of failure (FoF) are measured, as well as the net hope (NH) and the self-efficacy belief (SEB).

To respect the priority of data management, the main variables were reduced by five items so that the short version of the AMS could check the hope for success, and another five times for the fear of failure. This short version also demonstrates good-to-very good validity and reduces the total number of items, which is expected to encourage athletes to participate (α = 0.89). From the SMT the categories of flexibility, self-discipline, confidence, endurance and goal setting were under a special point of view.

## 3. Results

To understand the value of the findings of the study, firstly the group differences related to gender-specific factors were highlighted (see Table 1). It is imperative to elucidate any potential disparities in the results obtained at t_1_ between the two genders. This is essential to ascertain whether there is a need for enhanced comprehension to facilitate optimized support in the dual-career context.

As demonstrated in Table 1, there are partially significant differences between the genders with regard to fear of failure and confidence, exhibiting a small effect size ranging from 0.175 to 0.25. It is imperative that these results are given due consideration in order to formulate subsequent recommendations.

To prove the main goal of the study, the following Table 2 shows differences between the groups divided by different levels of performance.

The following correlation matrix provides a comprehensive overview of the main interdependencies between all variables measured at t_1_.

The final objective of Table 3 is to identify any potential correlations between the final success (performance level) and the variables, whereby these were found for the variables of self-confidence, goal setting and discipline. The results of the study indicate a moderate-to-strong correlation (*r* = 0.102–0.128, *p* = 0.001–0.01) between these variables and the success of the project. It is evident that these relationships are crucial for understanding the importance of supporting special issues for athletes and creating additional coaching opportunities.

In consideration of the fact that group no. 5 comprises a mere six athletes, a consequence of the elevated caliber of their sporting accomplishments, the descriptive statistics are exhibited. Nevertheless, these athletes are incorporated into the Kruskal–Wallis test analysis with group no. 4. This is the rationale behind the presentation of data in four groups in Table 4.

**Figure 2:**
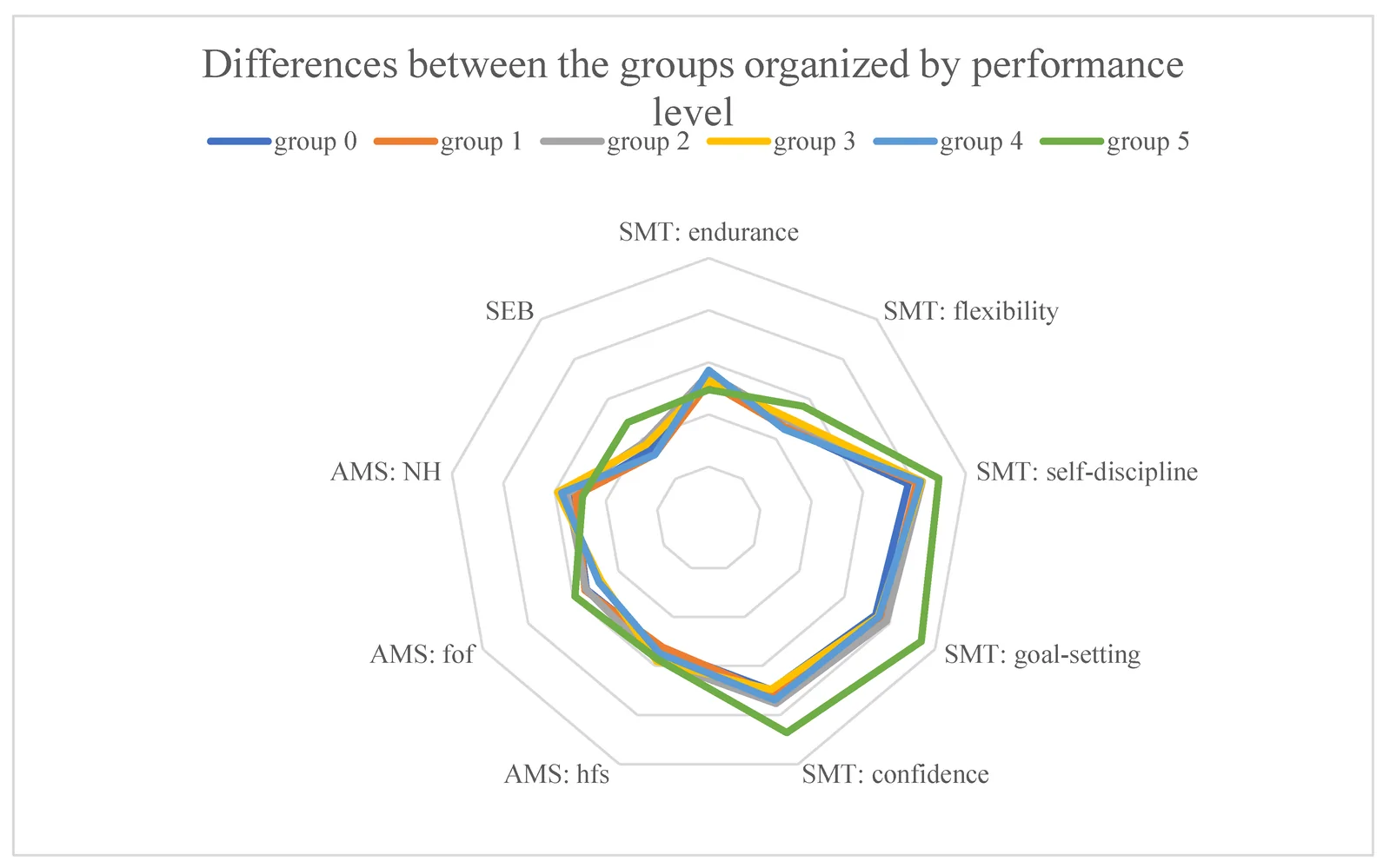
Different results for the main variables between the groups organized by performance level (0–5).

A comparison of all five groups (see Figure 2) shows significant differences in self-discipline (*p* = 0.010), goal setting (*p* = 0.013) and confidence (*p* = 0.029). In order to facilitate a more comprehensive and nuanced understanding of the findings, the post hoc results are exclusively presented for the group comparisons that exhibited significant differences, with some minor effect sizes ranging from 0.08 to 0.14.

### Comparison of Profiles

The objective is to create a profile of the athletes and then compare this profile (comprising the eight variables) with the athletes who could not be assessed during the sports entrance test. The findings of the study demonstrated that there were significant differences between the profiles of all groups when compared to the PK athletes. As outlined in the Methodology section, the test scales within a profile are standardized (e.g., standard scores) and their reliability is established, enabling changes in or comparisons of profiles to be analyzed using the Chi-squared distribution of profile scores. This methodical strategy is employed to compare groups of varying sizes, a key consideration when analyzing top international athletes, where participant numbers are typically limited.

The FoF, HfS and flexibility variables have low r_tt_ values (0.60–0.66), so a second calculation was performed to check whether the inclusion of these variables influences the overall result (see Table 5).

A significant correlation was identified between group affiliation and the variable values recorded at t_1_ for all variables, with small-to-medium effect sizes (*V* = 0.16–0.57).

## 4. Discussion

The findings of this study suggest that, on the one hand, a psychological profile at time point t_1_ can already indicate the extent of future career prospects. Conversely, it also highlights which areas should be prioritized. The study’s findings indicate that categories such as confidence, goal setting and self-discipline should be given due consideration. The analysis of gender-specific results indicates that a systematic approach to providing support regarding confidence and the management of fear of failure may enhance the psychological well-being of female athletes. The findings demonstrate significant disparities between the genders in this regard. It is logical to conclude that group 5 could have included more elite athletes. This could be considered a limitation of the study, as the general information about these athletes is intended to allow general conclusions to be drawn regarding the support of all talented athletes. Furthermore, it is important to note that the survey is only applicable to track and field athletes, and differences from athletes in other sports are to be expected. A comparison between team and individual sports could also be particularly interesting.

In response to the initial question regarding the support of mental skills at an early age, it is important to note the role of long-term support involving regular goal setting discussions. It is vital that confidence is fostered within the school support framework, and that positive reinforcement is used as a key method for achieving this. The coach’s communication, for example, influences athletes’ learning performance, so training courses for coaches and teachers on optimizing communication with athletes should be offered (Schröder, 2025a).

When considering the various psychological profiles at the commencement of a sports school career, as well as the broad spectrum of motivations, it is essential to establish a foundation for personalized support in day-to-day life at sports schools. Furthermore, these profiles change over time, so the profile trajectory and the individual profile parameters should be examined regularly (Pospeschill, 2025).

The objective of fostering mental strength is to develop the psychological abilities and skills that help athletes cope with stress and, ultimately, perform better (Denovan et al., 2024; Drinkwater et al., 2019). The development of the psychological constructs mentioned above is intended to boost self-confidence, thereby increasing the likelihood of sporting success (Hays et al., 2009; Feltz et al., 2009). High self-confidence enables a better assessment of challenging competitive situations, thereby protecting the athlete from inhibitory processes caused by emotions that are difficult to regulate, and which might otherwise be perceived as negative (Hanton et al., 2004; Mellalieu et al., 2006). Vealey (2001) attributes the sources from which athletes derive their self-confidence to the performance level, the self-regulation level and the social climate. It is evident that athletes who possess both a sense of anxiety and self-assurance are particularly adept at achieving high levels of performance. This finding is further substantiated by the results pertaining to the fear-of-failure motive in the present study (Hays et al., 2009).

In a systematic review incorporating data on a total of 3.711 athletes from 15 countries and 24 sports, Lochbaum et al. (2022) examined the relationship between self-confidence and sporting performance. The results show that there are weak-to-moderate correlations (up to *r* = 0.035), with moderating effects observed in relation to gender and sport type (team and individual sports). The strongest correlations were found for individual sports (*r* = 0.029) and male athletes (*r* = 0.034).

These findings emphasize the significance of early, long-term support, particularly support that is tailored to the specific needs of each group, as provided by established support systems at elite sports schools. In this context, Plakona et al. (2014) emphasize the significance of cultivating specific personality traits for optimal competition preparation. The authors explicitly address key competencies such as diligence, perseverance, determination, a sense of responsibility, emotional stability, self-control, self-confidence, composure and competitiveness. In this context, the strengthening of self-confidence is intended primarily to enable the aforementioned psychological personality traits to be maintained stably even in the face of adversity, and thus, for example, to remain unaffected by the influence of the opponent or the competition conditions.

The development and alignment of individual personality traits depend largely on the goals to be achieved, which should be tailored specifically to the athletes in order to ensure the greatest possible sporting success (Healy et al., 2018). It has become clear that goal setting, a topic that has been well researched since the 1980s, is of great importance. In this regard, the distinction between performance and process goals is significant because authors have demonstrated that these have different effects on athletes. Williamson et al. (2024) demonstrated in their systematic review that both types of goal setting have positive effects on sporting performance (*d* > 0.43), but that process goals have a significantly greater influence (*Q* = 4.77, *p* = 0.029). It is evident that the implementation of both short-term goals and a combination of short-term and long-term goals has resulted in a substantial enhancement in performance (*d* ≥ 0.43). A key finding is that short- and medium-term goals have a greater influence on sporting performance than long-term goals. Overall, the implementation of goal setting resulted in a positive impact on sporting performance across all groups; however, the most significant effects were observed in women (*d* = 1.50) and adolescents, who exhibited significantly superior results in comparison to adults (*Q* = 10.34, *p* = 0.001) (Williamson et al., 2024).

Achieving these goals, particularly in the context of a long-term school sports career, requires a level of self-discipline that enables athletes to successfully overcome even challenging situations.

In this context, Pujianto et al. (2024) highlight that particularly high levels of performance motivation were observed among 402 school-age athletes across 16 sports, with an average age of 15.9 years. Research indicates that these school athletes find it easier to maintain a sustained commitment to training, to believe in victory and to formulate clear goals (Ozrudi & Matmask, 2019; Pujianto et al., 2024).

Tedesqui and Young (2017) demonstrated that increased self-discipline among 244 athletes led to a greater willingness to train and also reduced the risk of increased thoughts of dropping out, thereby ultimately preventing premature career termination. These findings underscore the pivotal role that optimized support from the sports school can play. On the one hand, it can set clear goals, fostering self-discipline and mitigating the risk of attrition, which in turn is likely to substantially boost the chances of sporting success.

The link between academic and sporting education can be demonstrated in a number of ways; in a study of secondary school pupils, Subia (2019) found strong correlations between academic and sporting performance (*r* = 0.381, *p* < 0.01). This indicates that as academic performance improves, there is a corresponding increase in the participation of respondents in school sports. In an ideal sports school program, these effects should be attainable on both sides.

The concept of utilizing profiles created according to performance-based groups is not a novel one, and it exemplifies how certain profiles can be employed to optimize the management of specific influences in sport. As demonstrated in the study by Rose et al. (2023), certain profiles of endurance athletes cope with stress differently. This is due to low levels of stress resilience, which carry risks in both the mental and physical domains (Arnold et al., 2017; Ivarsson et al., 2017). It is important to note that younger athletes in particular need to acquire strategies for coping with stress, as they do not yet possess the experience and skills of older athletes in this regard and therefore tend to cope with stress in a negative manner. This underscores the importance of elite sports academies in fostering these skills from an early age and over the long term.

## Figures and Tables

**Table 1:** Gender-specific descriptive statistics incl. mean, SD of the main variables (t_1_) of the study and *t*-test.

Group	Male			Female			*t*-Test			
Variables/Statistics	Mean	*N*	SD	Mean	*N*	SD	df	*T*	*p*	Cohen’s *d*
Endurance	29.31	308	4.78	29.20	347	5.12	653	−0.27	0.78	−0.021
Flexibility	24.90	308	3.52	25.18	347	3.68	653	0.975	0.33	−0.076
Self-discipline	32.41	308	5.16	32.45	347	5.67	653	0.086	0.932	0.007
Goal setting	35.46	308	5.62	34.81	347	5.28	653	−1.53	0.13	−0.120
Confidence	35.45	308	5.62	34.80	347	5.28	653	−3.12	0.001	−0.250
Hope for success	34.21	308	5.5	32.92	347	5.26	653	−1.56	0.110	−0.125
Fear of failure	10.52	308	2.34	10.21	347	2.54	653	2.24	0.026	0.175
Net hope	4.1	308	2.68	4.61	347	3.9	653	−2.44	0.015	−0.191
SEB	27.87	263	3.59	27.47	283	3.92	544	−1.24	0.215	0.106

**Table 2:** Descriptive statistics incl. mean and SD of the main variables of the study for the different performing groups.

Group	Variable	Endurance	Flexibility	Self-Discipline	Goal Setting	Confidence	HfS	FoF	NH	SEB
0	Mean	29.10	24.90	31.78	34.56	33.03	10.26	4.41	5.84	27.49
	N	370	370	370	370	370	370	370	370	316
	SD	4.69	3.70	5.54	5.34	5.31	2.41	2.93	4.39	3.71
1	Mean	29.07	25.01	32.64	35.49	33.61	10.07	4.34	5.73	27.37
	N	91	91	91	91	91	91	91	91	75
	SD	5.45	3.43	5.41	6.34	5.23	2.47	2.57	3.88	3.72
2	Mean	29.59	25.35	33.48	35.91	34.45	10.69	4.46	6.22	28.01
	N	113	113	113	113	113	113	113	113	92
	SD	5.14	3.62	5.12	5.12	4.46	2.27	3.01	4.35	3.59
3	Mean	28.93	25.60	33.36	35.17	33.13	10.63	3.73	6.90	28.41
	N	30	30	30	30	30	30	30	30	22
	SD	4.94	3.40	5.08	6.16	6.32	2.97	2.91	4.82	3.95
4	Mean	29.82	25.21	33.00	35.09	34.13	10.43	3.65	6.78	27.31
	N	23	23	23	23	23	23	23	23	16
	SD	4.45	3.56	4.85	4.51	4.74	2.80	2.55	4.03	4.71
5	Mean	29.33	26.50	35.50	40.67	37.83	10.50	5.16	5.33	30.00
	N	6	6	6	6	6	6	6	6	3
	SD	7.36	2.07	3.78	1.21	2.92	1.97	3.92	5.12	2.00
Total	Mean	29.25	25.05	32.43	35.04	33.53	10.36	4.37	5.98	27.61
	N	633	633	633	633	633	633	633	633	524
	SD	4.96	3.61	5.43	5.49	5.20	2.45	2.92	4.36	3.73

**Table 3:** Correlation of all variables of t_1_.

Variable			1	2	3	4	5	6	7	8	9
1	SMT: Endurance	Pearson Correlation	1								
		Sig. (2-tailed)									
2	SMT: Flexibility	Pearson Correlation	0.275 **	1							
		Sig. (2-tailed)	<0.001								
3	SMT: Self-discipline	Pearson Correlation	0.507 **	0.368 **	1						
		Sig. (2-tailed)	<0.001	<0.001							
4	SMT: Goal setting	Pearson Correlation	0.333 **	0.451 **	0.443 **	1					
		Sig. (2-tailed)	<0.001	<0.001	<0.001						
5	SMT: Confidence	Pearson Correlation	0.400 **	0.439 **	0.427 **	0.669 **	1				
		Sig. (2-tailed)	<0.001	<0.001	<0.001	<0.001					
6	AMS: HfS	Pearson Correlation	0.438 **	0.304 **	0.388 **	0.368 **	0.446 **	1			
		Sig. (2-tailed)	<0.001	<0.001	<0.001	<0.001	<0.001				
7	AMS: FoF	Pearson Correlation	−0.375 **	−0.164 **	−0.256 **	−0.131 **	−0.273 **	−0.305 **	1		
		Sig. (2-tailed)	<0.001	<0.001	<0.001	<0.001	<0.001	<0.001			
8	AMS: NH	Pearson Correlation	0.497 **	0.281 **	0.387 **	0.293 **	0.432 **	0.764 **	−0.846 **	1	
		Sig. (2-tailed)	<0.001	<0.001	<0.001	<0.001	<0.001	<0.001	<0.001		
9	SEB	Pearson Correlation	0.438 **	0.326 **	0.383 **	0.425 **	0.595 **	0.528 **	−0.277 **	0.479 **	1
		Sig. (2-tailed)	<0.001	<0.001	<0.001	<0.001	<0.001	<0.001	<0.001	<0.001	
10	Performance level	Pearson Correlation	0.031	0.062	0.128 **	0.102 **	0.102 **	0.052	−0.035	0.053	0.057
		Sig. (2-tailed)	0.443	0.117	0.001	0.010	0.010	0.190	0.383	0.186	0.194

** The correlation is significant at the 0.01 level (two-tailed).

**Table 4:** Results of the analysis of the Kruskal–Wallis test related to the different performance groups (0–4).

Variable	Kruskal–Wallis Test						Post Hoc for Sig. Differences of the Groups				
	*n*	df	Z	*p*	η^2^	f	Variables	Groups *	*p*	Z	*r*
Endurance	655	4	1.129	0.890	−0.004	0.06	Self- discipline	0–1	0.029	−2.188	0.10
Flexibility	655	4	1.654	0.799	−0.003	0.05		0–2	0.003	−2.997	0.14
Self-discipline	655	4	13.257	0.010	0.014	0.12	Goal setting	0–1	0.006	−2.759	0.13
Goal setting	655	4	12.717	0.013	0.013	0.11		0–2	0.011	−2.528	0.12
Confidence	655	4	10.777	0.029	0.010	0.10	Confidence	0–1	0.076	−1.774	0.08
AMS: HfS	655	4	4.187	0.381	0.000	-					
AMS: FoF	655	4	2.633	0.621	−0.002	0.04		0–2	0.007	−2.682	0.12
AMS: NH	655	4	2.851	0.583	−0.002	0.04		0–4	0.054	−1.930	0.10
SEB	546	4	4.351	0.361	0.000	-					

* The comparison of the other groups did not show any significant difference.

**Table 5:** Profile comparisons of the prospective squad group with groups 0–4 at t_1_.

Comparison Between the Groups and the Top Performers from Group No. 5		Profile Comparison According to Kristof (Group Profiles Based on the Eight Variables *)					
		df	*N*	r_tt_	χ^2^	*p*	Cramer’s *V*
**5**	0	8	376	0.60–0.85	43.2	0.001	0.17
		5		0.71–0.85	40.88		0.16
	1	8	97	0.60–0.85	39.36	0.001	0.32
		5		0.71–0.85	36.35		0.31
	2	8	119	0.60–0.85	21.84	0.001	0.21
		5		0.71–0.85	20.58		0.21
	3	8	36	0.60–0.85	26.21	0.001	0.43
		5		0.71–0.85	21.65		0.39
	4	8	29	0.60–0.85	37.35	0.001	0.57
		5		0.71–0.85	33.52		0.54

* df 5, profile without variables FoF, HfS and flexibility.

## Data Availability

The data that support the findings of this study are available on request from the corresponding author (Sebastian Schröder). The data are not publicly available due to restrictions, e.g., containing information that could compromise the privacy of research participants.

## Abbreviations

The following abbreviations are used in this manuscript:

ESS	Elite sports schools
SEB	Self-efficacy belief
HfS	Hope for success
FoF	Fear of failure
NH	Net hope
SMT	Sport motivation test
AMS	Achievement motive scale
NK	Nationalkader (national squad)
PK	Perspektivkader (prospective squad)
