# Correction: Schröder et al. (2026). Mental Skills Training: An Often-Overlooked Aspect of Preparation for Future High-Performing Athletes in Sports Schools. *Behavioral Sciences*, *16*(7), 1109

**DOI:** 10.3390/bs16081434

**Published:** 2026-08-20

**Authors:** Sebastian Schröder, Christine Stucke, Tabea Linkohr, Melanie Schulz

**Affiliations:** 1 Department III Philology, Philosophy, Sports, Otto-von-Guericke-Universität, Magdeburg 1, 39104 Magdeburg, Germany; 2 Faculty of Culture, Media, Psychology, Macromedia University of Applied Science, 04105 Leipzig, Germany; 3 Leichtathletik-Verband Sachsen-Anhalt e. V., 06120 Halle, Germany

**Change of Corresponding Author and Email Address.**

In the original publication Schröder et al. (2026), the corresponding author was incorrectly designated as Melanie Schulz. The corresponding author has been changed to Sebastian Schröder.

The email address for Melanie Schulz has been updated from bildung@lvsa.de to landestrainer@lvsa.de.

The authors state that the scientific conclusions are unaffected. This correction was approved by the Academic Editor. The original publication has also been updated.

## References

[B1-behavsci-16-01434] Schröder S., Stucke C., Linkohr T., Schulz M. (2026). Mental skills training: An often-overlooked aspect of preparation for future high-performing athletes in sports schools. Behavioral Sciences.

